# Sequential Extraction of Soluble and Insoluble Alpha-Synuclein from Parkinsonian Brains

**DOI:** 10.3791/53415

**Published:** 2016-01-05

**Authors:** Rina Bandopadhyay

**Affiliations:** ^1^Reta Lila Weston Institute of Neurological Studies, UCL Institute of Neurology

**Keywords:** Neuroscience, Issue 107, alpha-synuclein, aggregation, insoluble alpha-synuclein, Lewy body, post-mortem brain, ultracentrifugation, Parkinson's disease, immunoblotting.

## Abstract

Alpha-synuclein (α-syn) protein is abundantly expressed mainly within neurons, and exists in a number of different forms - monomers, tetramers, oligomers and fibrils. During disease, α-syn undergoes conformational changes to form oligomers and high molecular weight aggregates that tend to make the protein more insoluble. Abnormally aggregated α-syn is a neuropathological feature of Parkinson's disease (PD), dementia with Lewy bodies (DLB) and multiple system atrophy (MSA). Biochemical characterization and analysis of insoluble α-syn using buffers with increasing detergent strength and high-speed ultracentrifugation provides a powerful tool to determine the development of α-syn pathology associated with disease progression. This protocol describes the isolation of increasingly insoluble/aggregated α-syn from post-mortem human brain tissue. This methodology can be adapted with modifications to studies of normal and abnormal α-syn biology in transgenic animal models harbouring different α-syn mutations as well as in other neurodegenerative diseases that feature aberrant fibrillar deposits of proteins related to their respective pathologies.

**Figure:**
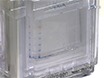


## Introduction

One of the key pathological features common amongst several neurodegenerative diseases is the formation of abnormal protein aggregates that occurs in a protein/disease specific manner^1^. Thus, there are the characteristic extraneuronal amyloid-beta plaques and aggregated hyperphosphorylated tau deposits within neurons in Alzheimer's disease (AD); the aggregated α-syn deposits in Lewy Bodies (LBs) which are intraneuronal inclusions, and also within dystrophic neuronal processes called Lewy neurites (LNs) in PD, PD dementia and Dementia with Lewy bodies (DLBs)^2-4^. Abnormal α-syn deposits are also seen in hallmark lesions of glial cytoplasmic inclusions in MSA^5^. In Huntington's disease, there are the characteristic Poly-Q deposits, and abnormal Tar DNA binding protein-43 and fused in sarcoma proteins (FUS) are deposited in frontotemporal dementias. Importantly for PD, α-syn gene mutations have been found to cause autosomal dominant PD^6-11^. Additionally, α-syn gene triplication^12^ and duplication^13^ also causes familial PD thus linking increased α-syn expression to the pathomechanisms of PD. Notably, both sporadic and familial PD cases harbour abnormal deposits of α-syn pathology^14^. Current available treatments for PD are only palliative and have no effect on halting or delaying the inexorable disease progression.

α-syn is abundantly expressed in the human brain and makes up about 1% of the total brain protein. It is present more specifically within the neurons but can exist albeit in lower amounts within glial cells. A contentious point is the native structure of α-syn which can exist as a random monomer^15^ or as a folded tetramer^16^. During disease, α-syn changes its conformation that allows it to get misfolded and this process is thought to be the cause of disease pathogenesis. Despite several years of investigations into the pathophysiological aspects of α-syn and disease, the exact causes of disease mechanisms have remained elusive^14^.

Studies using post-mortem analysis of Parkinsonian brains have thus far provided important clues regarding normal and abnormal biology of α-syn both in sporadic PD and also PD associated with mutations in the LRRK2 gene^17-22^. Here, a biochemical extraction protocol (see scheme presented in **Figure 1**) for increasingly insoluble α-syn deposits from human post-mortem PD brain tissue that harbour α-syn aggregates to varying degrees has been described. This technique can also be adapted with modifications in buffer compositions to study aggregate proteins from other neurodegenerative diseases^23,24^ and also from transgenic animal brain tissue^25-27^. The main consideration for the adaptations are the differences in solubility and the total amounts of the respective proteins some of which are mainly nuclear and may need high-salt buffers for optimal extraction as shown for FUS^28^.

## Protocol

Post-mortem brain tissue was donated to Queen Square Brain Bank for neurological disorders, University College London, Institute of Neurology using ethically approved protocols and stored for research under a license issued by the human tissue authority (HTA) UK (no. 12198). See list of cases used for this protocol (**Table 1**).

### 1. Preparation of Buffers

1. Prepare 1X TBS (Tris-buffered saline) buffer:
  1. Prepare 10X TBS as stock solution with 500 mM Tris-HCl pH 7.4 and 1,500 mM NaCl.
    1. Weigh out 60.5 g Tris-Cl pH 7.6 and 87.6 g of NaCl in 800 ml of high purity water. Adjust pH with 1 M HCl and make the final stock volume to 1 L.
  2. Prepare the final concentration of 1X TBS by diluting the stock 10 times in distilled water.
  3. Add protease inhibitors (PI), (1 tablet per 50 ml buffer) and Phos-stop phosphatase inhibitor tablets (1 tablet per 10 ml of buffer) to abrogate the effects of non-specific enzymatic actions of proteases and phosphatases. Note: Here we have used the enzyme inhibitor tablets from Roche but equally other commercially available inhibitors can be used according to the manufacturer's instructions.
2. Prepare TBS-SDS buffer by adding 5% w/v Sodium dodecyl sulphate (SDS) to 1X TBS (pH 7.4) buffer. Heat the solution to 60 °C with constant stirring using a magnetic stirrer to solubilize SDS.
3. Prepare TBS-SDS-Urea buffer by adding urea to a final concentration of 8 M w/v to 1X TBS-SDS buffer (pH 7.4). Heat the solution to 60 °C with constant stirring on a magnetic stirrer to dissolve urea.

### 2. Sample Homogenization and Differential Ultracentrifugation

Note: The following part of the work involves handling of human brain tissue according to the rules of HTA UK. Local standard operating procedures are followed at all times. Brain samples are dissected from frozen brain blocks and tissue material homogenized in a microcabinet maintained at negative pressure. Disposable gowns gloves, face-masks, over-shoe protectors and safety goggles are worn throughout the procedure. Human tissue waste is discarded after autoclaving at 121 °C for 20 min for safe disposal. Sharps (scalpel blades) are disposed in an appropriate sharps container for containment and safe disposal.

1. Take a chunk of frozen human tissue (approximately 0.5 g in weight from the basal ganglia region) and rapidly mince into small fragments (approximately 1 mm^2^) on ice using a small petri-dish and a sterile scalpel blade. Use a separate petri-dish and scalpel blade for each sample. Collect the minced tissue in a 15 ml tube and add 10 volumes of ice-cold 1XTBS buffer to the tube.
2. Homogenize the samples using a mechanical homogenizer at 20,000 rpm for 10 sec. Cool on ice for 2 min. Repeat this step three times. Note: This is done so that the samples are not heated up while homogenizing.
3. Clarify by centrifugation at 1,000 x g for 5 min at 4 °C. Discard pellet of non-homogenized material.
4. Take supernatant (crude homogenate), add to polycarbonate centrifuge tubes (size of tube depends on rotor size) and balance carefully. Centrifuge at 100,000 x g for 1 hr at 4 °C.
5. Retain supernatant as this is the TBS-soluble fraction.
6. Wash pellet twice in five volumes of 1X TBS buffer and centrifuge each time at 100,000 x g for 15 min at 4 °C. Discard the supernatants.
  1. Resuspend the final pellet by sonicating the pellet for 10 sec at 20 KHz in approximately 5 volumes of 1X TBS-SDS at RT. Note: Before addition of the SDS containing buffer samples should be brought at 10 °C to avoid SDS precipitation.
  2. Ultracentrifuge at 100,000 x g for 30 min at 25 °C.
7. Retain supernatant as this is the SDS soluble fraction.
  1. Wash pellet twice in 5 volumes of 1X TBS-SDS buffer at RT. Centrifuge each time at 100,000 x g for 15 min at 25 °C.
  2. Solubilize the final pellet in 50 μl of 1X TBS-SDS-UREA buffer using a sonicator set at 20 KHz for 10 sec to attain complete solubilization; this is termed the urea soluble fraction.
8. Assay each fraction for total protein content using a commercial protein assay kit according to manufacturer's protocol. Dilute TBS-SDS-Urea samples 1:1 with 1X TBS buffer to allow compatibility with protein assay reagents. Freeze samples in small aliquots (20 μl) at -80 °C to reduce freeze-thaw cycles. Note: Adding 10% glycerol to samples is recommended for long-term storage at -80 °C.

### 3. Immunoblotting of Samples and Analyzing Results

1. Run samples from TBS, TBS-SDS and TBS-SDS-Urea extracts on 10-well 4-12% Bis-Tris polyacrylamide gel with MOPS as running buffer using standard techniques. See Gallagher and Chakravarti (2008)^29^ for a detailed western blot protocol.
2. Load 10 µg of protein from each sample onto each lane along with a molecular weight marker for TBS and SDS and urea fractions.
3. Run gel at 200 V for 1 hr or until the blue dye from loading buffer has reached the bottom of the gel.
4. Transfer proteins from gels electrophoretically onto nylon membranes^28^, pre-wetted first in 100% methanol and then into transfer buffer containing 20% methanol. Provide an identification mark on the blot to determine the protein side and the orientation of the Molecular-Weight size markers.
  1. Sandwich the gel with the membrane alongside wet filter paper. Correct placement of membrane is crucial with the membrane between the gel and the anode. Perform the transfer for 2 hr at 40 V.
  2. Make up 1X PBS-tween (1X PBS-T) buffer: Dissolve 1 tablet of PBS in 200 ml water to yield 0.01 M phosphate buffer, 0.0027 M potassium chloride and 0.137 M sodium chloride, pH 7.4.
  3. Block the membrane in 5% BSA solution in 1X PBS-T for 30 min with shaking at 70 rpm. This prevents non-specific binding of primary antibody to the membrane.
5. Probe the protein blot with 6 ml of α-syn primary antibody Syn-1 (BD Biosciences; mouse monoclonal antibody) at 1:750 dilution (O/N at 4 °C) followed by a series of washes with 1X PBS-T (3 x 5 min each time)^28^.
6. Incubate the blot with the appropriate HRP conjugated secondary antibody at 1:2,000 dilution for 30 min. Perform a series of washes (3 x 5 min each time) with 1X PBS-T.
  1. Immerse blots in enhanced chemiluminescence solution for 15 sec in a dark room. Cover blots with a cling film and place autoradiography films against the blot with protein side up in a light-proof cassette to capture the appropriate signals (appropriate size bands).
  2. Develop autoradiography films in an automated developer. Note: Blots can also be developed manually using developer and fixer from a commercial source in case an automated developer machine is not available.
7. Incubate the western blot with 6 ml of western blot stripping buffer for 10 min with constant shaking to strip the blot of α-syn antibody signal. Follow steps 3.43 through to 3.6.2 using beta-actin primary antibody (Mouse monoclonal) at 1: 8,000 dilution.
8. Scan and convert the final image into a Tiff image and measure densities using NIH ImageJ for quantitation purposes (a free downloadable software). Note: The software allows reading of densities of relative area of interest (the appropriate size bands) and the data can be expressed either as a ratio of α-syn density / β-actin (a house-keeping gene) as arbitrary units or on its own when a house keeping gene is not valid (as in the case of urea-soluble samples).

## Representative Results

TBS, SDS and Urea soluble proteins extracted from basal ganglia following the protocol shown in **Figure 1** were run on gels and immunoblotted with the Syn-1 mouse monoclonal α-syn primary antibody. The TBS soluble fractions displayed the presence of monomeric α-syn (~14 KDa species) in the PD and control cases examined (**Figure 2A**). The SDS soluble fractions also showed abundant monomeric α-syn in all three subtypes of cases studied as displayed in the low exposure blot (**Figure 2C**). The PD cases also showed higher molecular weight (MW) α-syn species as seen on the high exposure blot, which are likely to be oligomers (**Figure 2C**). The urea soluble fractions from PD cases showed elevated amounts of α-syn monomers, oligomers and aggregated species compared to control cases. The neocortical idiopathic PD cases demonstrate higher amounts of aggregated α-syn species, whilst the limbic cases show intermediate levels of insoluble α-syn. Both the SDS and urea fractions from idiopathic PD cases demonstrate variable levels of truncated α-syn products at 12 kDa and 6 kDa as described ^20^. In contrast, the control cases did not show any insoluble or aggregated α-syn (**Figure 2E**). Semi-quantitative density measures reflect the quantity of LB load as categoriesd using α-syn immunohistochemistry demonstrating the insoluble nature of aggregated α-syn (**Figure 2B**, **2D**, **2F**) in neocortical and limbic PD cases.


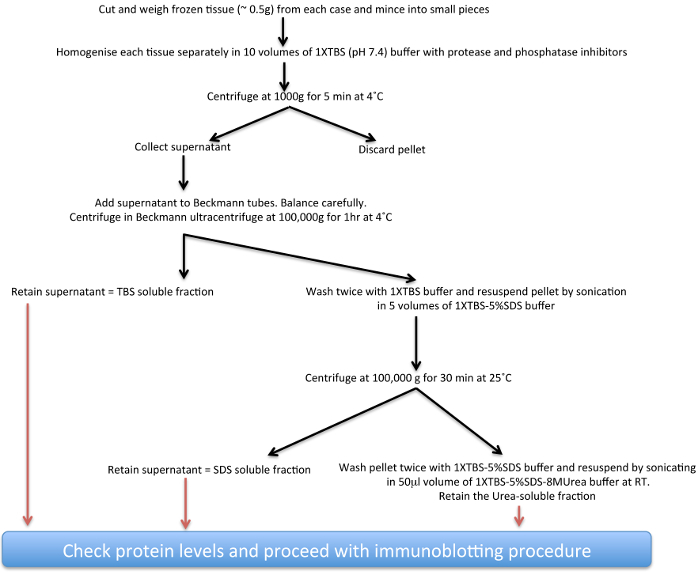
 **Figure 1. Schematic diagram of insoluble** **α** **-syn extraction procedure.** Please click here to view a larger version of this figure.


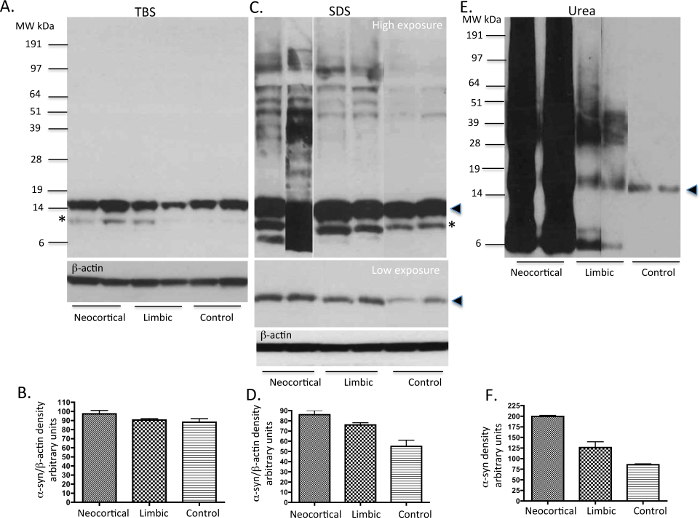
**Figure 2. Representative immunoblots of α-syn levels from PD and control human tissue.** TBS, SDS and urea soluble fractions from basal ganglia (a region that harbors α-syn pathology in PD cases) of idiopathic PD cases and neurologically normal (control) brains were analysed for the presence of α-syn using western immunoblot. 10 μg of protein was loaded onto each lane. In TBS fractions (**A**), mainly α-syn monomers are present in all cases. In SDS fractions (**C**), some oligomers of α-syn are seen along with monomers mainly in the PD tissue. In urea fractions (**C**), monomers, oligomers and aggregated α-syn are variably expressed in the PD cases reflecting their respective LB load. The neurologically normal control samples show the presence of only small amounts of monomeric α-syn. Please note possible degradation products of α-syn are seen in some of the cases with high LB load. The solid arrow head represents the monomeric form of α-syn. The * possibly represents a C-terminally truncated form of α-syn as previously described^20,26^. Please click here to view a larger version of this figure.

**Table:** 

**Case **	**Sex**	**age at death yrs**	**post-mortem delay (hrs)**	**pH of tissue **
Neocortical 1	M	82	40	6.3
Neocortical 2	F	75	35.3	6.4
Limbic 1	M	81	28.5	6.2
Limbic 2	F	79	36.5	6.2
Control 1	M	80	38	6.1
Control 2	F	83	32.5	6.3

**Table 1. Limited demographics of the cases used**

## Discussion

This article describes a biochemical protocol for extraction and examination of the differential solubility properties of monomeric, oligomeric and aggregated α-syn from diseased brains using denaturing gel running conditions. This is a well-established technique to study aggregated α-syn^17, 18, 20, 21 ^a protein that is normally soluble in its native form but gain amyloidogenic or increased aggregation properties with disease progression or with genetic mutations. Nonetheless, some groups have used slight variations in SDS concentrations in their buffers (8% or 10% instead of 5%)^21,22, 30^. The SDS is an anionic detergent and solubilises α-syn oligomers that can include membrane bound forms of α-syn, whereas urea, a chaotropic reagent denatures the insoluble aggregated and fibrillar or amyloidogenic forms of α-syn^20^. In this regard a systematic study by Paleologou et al^31^ examined the stability of α-syn oligomers and fibrils with varying concentrations of urea (6.5 - 8 M) or SDS (0.25-2%) and reported that oligomers stable in SDS concentrations but not with high concentrations of urea. Their FILA-1 antibody, specific for α-syn oligomers and fibrils detected higher concentrations of fibrils at 6.5 M urea concentrations under non-denaturing conditions. The buffer concentrations used in this protocol closely mirrors what has been described in Culvenor *et al*. (1999)^32^.

The anatomical brain regions for biochemical extraction of insoluble α-syn should be carefully selected and this should reflect α-syn LB and LN load depending on disease progression^33,34^. The cases used here are basal ganglia samples from the neocortical and limbic subtypes of PD reflecting late and intermediate stages of PD progression according to McKeith ctriteria^34^. At the Queen Square Brain Bank, one half of the brain is routinely fixed in formalin for histochemical analysis and the other half is carefully dissected into different anatomical regions and flash frozen and stored at -80 ^o^C freezers for further biochemical and DNA/RNA analysis studies. Following formalin fixation of the brains and dissection by neuropathologists, immunohistochemistry is performed using α-syn antibody and results archived. Also as a routine procedure, the pH of the brain tissue is measured upon arrival as a measure of agonal state of the brain tissue. This forms a firm basis for the quality of tissue preservation for biochemical analysis.

Our data here shows that there are more SDS soluble α-syn monomer in PD cases compared to controls; in particular the neocortical PD cases has higher α-syn load compared to the limbic PD variety. The neocortical cases represent greater progression of PD α-syn pathology in the neocortical regions such as the frontal and parietal cortices^33,34^. The limbic PD cases have higher LB scores in the basal forebrain/limbic regions areas such as the amygdala, transentorhinal and the cingulate regions. For meaningful data and statistical analysis, one must run at least 4 cases from each cohort. Likewise we have not attempted statistical analysis of the blot data presented here.

It must also be noted that different antibodies may recognize different forms/composition of higher order α-syn oligomers and each may have its own relative sensitivity and preference. This is evident from the results of Tong *et al*.^29^ where they show that 4 different α-syn antibodies (Syn-1, SS, Onco and LB509) give changeable results at least for α-syn oligomers/aggregates. The α-syn monomers were recognized to similar extents by all 4 antibodies although these can have variation depending on whether the α-syn antibody epitope is located either in the N-terminal or C-terminal^29^. Similarly, specific antibodies for phospho-alpha synuclein determine distinct high-molecular weight α-synuclein species^20,21^. In the protocol described here, the highly characterized antibody Syn-1 has been used^19-21^_._

However, it is important to consider validating any new antibody on western blots through antibody preabsorption experiments and also overexpression and knockdown of the protein in cells.

It is important to consider other variations that have been applied by researchers to determine smaller fragments of α-syn and or unstable tetrameric α-syn forms. This could include mild-fixation of membranes with 0.4% PFA in PBS to retain truncated forms of α-syn^35^ or treatment of sample homogenates with cross linkers to stabilize tetramers^36^.

Accurate biochemical evaluation of proteins using post-mortem tissue requires minimization of enzymatic protein degradation and modification. It is therefore advisable to use tissue with the shortest post-mortem delays and /or select matched samples within the case cohorts. Immunohistochemistry using standard antibodies for α-syn immunohistochemistry should be performed prior to starting the isolation protocol. The extent of α-syn pathology is highly variable and can vary according to regions selected. It is advisable to select regions with abundant pathology as a positive control for optimal yield with every run. The use of protease inhibitors and if necessary phosphatase inhibitors to prevent undesired enzymatic degradation after release of intracellular proteases or phosphatases occurring during cell lysis and maintaining the samples at 4 °C is crucial.

It is important that all of the samples within the batch of experiments are handled evenly and consistently to avoid intra-user variations; multiple freeze-thaw cycles should be avoided as this may potentially retract post-translational modifications such as phosphorylation. A critical point to bear in mind when examining a large number of samples is that the data from immunoblots should be normalized to one sample, which should run in parallel to all other samples on the gels, and all data referenced to that sample. This is done to ensure normalization of immunoblot data between several blots. This is the method adopted in our recent study^22^.

It should be noted that in order to keep the background ECL low, the blots should not be allowed to dry out at any time during the western blot protocol. In addition, very long exposures (>10 min) on autoradiography films should be avoided as this will lead to saturation of ECL signal and will lead to inaccurate quantitation.

This above protocol is a relatively straightforward technique using common laboratory reagents and instruments and can be a valuable tool for investigating the pathophysiology of α-syn protein in PD research. This methodology can not only be extended with appropriate modifications to the study of α-syn biology in α-syn transgenic animals but also to other neurodegenerative diseases featuring abnormal deposits of aggregated proteins such as AD, MSA, DLB, HD and frontotemporaldementias. However the western blot data described here could also be validated using enzyme-linked immunosorbent assays using antibodies for specific forms of α-syn^31^.

## Disclosures

The author has nothing to disclose.

## References

[B0] Forman MS, Trojanowski JQ, Lee VM (2004). Neurodegenerative disease a decade od discoveries paves the way for therapeutic breakthroughs. Nat Med.

[B1] Spillantini MG, Schmidt ML, Lee VM, Trojanowski JQ, Jakes R, Goedert M (1997). A-synuclein in Lewy bodies. Nature.

[B2] Spillantini MG, Crowther RA, Jakes R, Hasegawa M, Goedert M (1998). Alpha-synuclein in filamentous inclusions of Lewy bodies from Parkinson's disease and dementia with Lewy bodies. Proc Natl Acad Sci.

[B3] Baba M (1998). Aggregation of alpha-synuclein in Lewy bodies of sporadic Parkinson's disease and dementia with Lewy bodies. Am J Pathol.

[B4] Ahmed Z (2012). The neuropathology, pathophysiology and genetics of multiple system atrophy. Neuroptahol Appl Neurobiol.

[B5] Ploymeropoulos MH (1997). Mutation in the alpha-synuclein gene identified in families with Parkinson's disease. Science.

[B6] Kruger R (1998). Ala30Pro mutation in the gene encoding alpha-synuclein in Parkinson's disease. Nat Genet.

[B7] Zarranz JJ (2004). The new mutation, E46K, of alpha-synuclein causes Parkinson and Lewy Body dementia. Ann. Neurol.

[B8] Proukakis C (2013). A novel alpha-synuclein missense mutation in Parkinson disease. Neurology.

[B9] Kiely A (2013). Synucleinopathy associated with G51D SNCA mutation: a link between Parkinson's disease and Multiple System atrophy?. Acta Neuropathol.

[B10] Lesage S (2013). G51D alpha-synuclein mutation causes a novel Parkinson-pyramidal syndrome. Ann Neurol.

[B11] Singleton AB (2003). Alpha-synuclein locus triplication causes Parkinson's disease. Science.

[B12] Chartier-Harlin MC (2004). Alpha-synuclein locus duplication as a cause of familial Parkinson's disease. Lancet.

[B13] Cookson MR, Hardy J, Lewis PA (2008). Genetic neuropathology of PD. Int J Clin Exp Pathol.

[B14] Fauvet B (2012). α-synuclein in central nervous system and from erythrocytes, mammalian cells, and. Escherichia coli exists predominantly as disordered monomer. J Biol Chem.

[B15] Bartels T (2011). α-synuclein occurs physiologically as a helically folded tetramer that resists aggregation. Nature.

[B16] Campbell BC (2000). Accumulation of insoluble alpha-synuclein in dementia with Lewy bodies. Neurobiol of Dis.

[B17] Campbell BC (2001). The solubility of alpha-synuclein in multiple system atrophy differs from that of dementia with Lewy bodies and Parkinson's disease. J Neurochem.

[B18] Miller DW (2004). Alpha-synuclein in blood and brain from familial Parkinson's disease with SNCA locus triplication. Neurology.

[B19] Anderson JP (2006). Phosphorylation of Ser-129 is the dominant pathological modification of alpha-synuclein in familial and sporadic Lewy body disease. J Biol Chem.

[B20] Zhou J (2013). Changes in the solubility and phosphorylation of alpha-synuclein over the course of Parkinson's disease. Acta Neuropathol.

[B21] Mamais A (2013). Divergent solubility and aggregation properties in G2019S LRRK2 Parkinson's disease brains with Lewy Body pathology compared to idiopathic cases. Neurobiol of Dis.

[B22] Lashley T (2011). A comparative clinical, pathological, biochemical and genetic study of fused in sarcoma proteinopathies. Brain.

[B23] Brelstaff J (2011). Transportin-1: a marker of FTLD-FUS. Acta Neuropathol.

[B24] Kahle PJ (2002). Hyperphosphorylation and insolubility of alpha-synuclein in transgenic mouse oligodendrocytes. EMBO reports.

[B25] Nuber S (2013). A progressive dopaminergic phenotype associated with neurotoxic conversion of α-synuclein in BAC-transgenic rats. Brain.

[B26] Tofaris GK (2006). Pathological changes in dopaminergic nerve cells odf the substantia nigra and olfactory bulb in mice transgenic for truncated human a-synuclein (1-120): implications for Lewy Body disorders. J Neurosci.

[B27] Neumann M (2011). FET proteins TAF15 and EWS are selective markers that distinguish FTLD with FUS pathology from amyotrophic lateral sclerosis with FUS mutations. Brain.

[B28] Gallagher S, Chakravarti D (2008). Immunoblot analysis. J Vis. Exp.

[B29] Tong J (2010). Brain alpha-synuclein accumulation in multiple system atrophy, Parkinson's disease and progressive supranuclear palsy: a comparative investigation. Brain.

[B30] Paleologou KE (2009). Detection of elevated levels of soluble α-synuclein oligomers in post-mortem brain extracts from patients with dementia with Lewy bodies. Brain.

[B31] Culvenor JG (1999). Non-Aβ component of Alzheimer s disease Amyloid (NAC) revisited. American Journal of Pathology.

[B32] Braak H (2003). Staging of brain pathology related to sporadic Parkinson's disease. Neurobiol Aging.

[B33] McKeith IG (2005). Diagnosis and management of dementia with Lewy bodies: third report of the DLB consortium. Neurology.

[B34] Lee BR, Kamitani T (2011). Improved immunodetection of endogenous α-synuclein. PLoS ONE.

[B35] Newman AJ (2013). A new method for quantitative immunoblotting of endogenous α-synuclein. PLoS ONE.

